# The mediating role of organizational commitment between workplace bullying and turnover intention among clinical nurses in China: a cross-sectional study

**DOI:** 10.1186/s12912-023-01547-8

**Published:** 2023-10-06

**Authors:** Guili Xia, Yi Zhang, Ling Dong, Fengtao Huang, Yao Pu, Jiang Luo, Yi-ping Chen, Zhengxia Lei

**Affiliations:** 1 Department of Gastroenterology, Shenzhen Hospital, Southern Medical University, Shenzhen, Guangdong China; 2 Hunan Key Laboratory of Oral Health Research & Xiangya Stomatological Hospital & Xiangya School of Stomatology, Central South University, Changsha, Hunan 410008 China

**Keywords:** Nurses, Workplace bullying, Organizational commitment, Turnover intention

## Abstract

**Background:**

Workplace bullying experienced by clinical nurses is a critical and pervasive issue that not only detrimentally impacts nurses but also poses a significant threat to the overall quality of nursing services and patient care. This study aimed to determine the mediating role of organizational commitment in the relationship between workplace bullying and turnover intention among clinical nurses in China.

**Methods:**

Participants were recruited from 40 hospitals in various provinces of China from December 2, 2021 to February 25, 2023, using convenience sampling. After obtaining hospital ethical approval and participants’ informed consent, clinical nurses (n = 585) from different nursing departments in different hospitals completed the questionnaire. The Socio-demographic Questionnaire, Negative Acts Qestionnaire, Chinese Workers’ Organizational Commitment Scale and Turnover Intention Questionnaire were used to collect general demographic data of nurses and assess workplace bullying they experienced, their level of organizational commitment and turnover intention. Descriptive statistics, Pearson correlation analyses and structural equation model were adopted to analyze the data.

**Results:**

Pearson’s correlation analysis showed that that workplace bullying was significantly negatively correlated with organizational commitment (r = − 0.512, P<0.01) and significantly positively correlated with turnover intention (r = 0.558, P<0.01), organizational commitment was significantly negatively correlated with turnover intention (r = − 0.539, P<0.01). Mediation analysis indicated organizational commitment partially mediated the association between workplace bullying and turnover intention. The total effect (β = 0.69) of workplace bullying on turnover intention consisted of its direct effect (β = 0.41) and the indirect effect mediated through organizational commitment (β = 0.280), with the mediating effect accounting for 40.58% of the total effect.

**Conclusion:**

Organizational commitment mediated the associations of workplace bullying and turnover intention. Therefore, healthcare organizations and nursing managers should develop appropriate strategies to enhance nurses’ organizational commitment in order to reduce their turnover intention.

## Introduction

### Workplace bullying

Workplace bullying is a serious social issue [1], which has a great impact on the development of nursing and nurses. Workplace bullying has been defined as “a situation where employees are consistently subjected to negative and aggressive words or behavior at work” [2]. Academics have reported on bullying in different research settings around the world and studies has shown that workplace bullying is more common and severe in nursing than in other professions [3, 4]. De Cieri et al. found that 42% of healthcare professionals, including nurses, had experienced bullying at work in the previous 12 months [5]. According to a cross-sectional study among clinical nurses in China, 68% of nurses had experienced workplace bullying in the previous 12 months [6]. For individual nurses, workplace bullying can negatively affect them both physically and psychologically. Studies have shown that nurses face physical health risks such as fatigue, angina [7, 8], hypertension and heart disease [9] as a result of workplace bullying. In addition, workplace bullying can lead to psychological problems such as anxiety, depression, post-traumatic stress disorder [10] and increased the risk of suicide among nurses [11]. For organizations, workplace bullying can lead to a large number of problems related to nurses’ work, such as reduced job satisfaction, poor job performance, diminished working relationships, burnout, increased nurse mobility and hindered organizational growth [1, 12, 13]. For patients, workplace bullying has the potential to compromise the delivery of high-quality nursing care, exert negative influences on nursing practice and patient outcomes, amplify risk factors for compromised patient safety, and potentially result in socioeconomic ramifications [14]. Therefore, workplace bullying needs more attention and solutions.

### Turnover intention

Turnover intention refers to “the desire of an individual to leave his or her current job within a certain period of time” [15], which is considered a cost-effective measure and a key predictor of turnover behavior, and is supported by a great deal experience and theory [16–19]. A survey of nurses working in general acute hospitals in Europe and the USA found that intentions to leave ranged from 14–49% [20]. It has been reported that approximately 40% of registered nurses in a Malaysia hospital intend to leave [21]. The departure of nurses means a loss of organizational value, and when the turnover intention becomes a reality, the costs associated with the recruitment, selection and integration of new nurses can be an expensive process for the organization [22]. Bullying is reported to be one of the factors that exacerbates turnover in the nursing workplace [23]. Previous studies have shown that workplace bullying is significantly positively correlated with nurse’ turnover intention, which is an international issue that needs attention [1, 24]. However, although the well-established link between workplace bullying and turnover intention, the mechanisms behind this relationship and its boundary conditions have not been fully explored [25].

### Organizational commitment

Organizational commitment is considered as one of the predictors of turnover intention. Organizational commitment is defined as “a state of mind or a psychological state of the relationship between the employee and the organization”. It is a description of the relationship between an organization and its members. Organizational commitment manifests itself in members accepting the values of the organization, being willing to work for the organization, and deciding to remain in or leave the organization [26]. Negative work outcomes such as nursing errors, poor quality of care and high turnover rates are associated with low levels of nurses’ organizational commitment [27]. Rodwell J [28] and Filipova A. A [29] found that hospital nurses who were bullied reported lower levels of commitment. Regarding the relationship between workplace bullying, organizational commitment and turnover intention, some researchers have explored the relationship between two of these variables individually, but these three variables have never been explored in the same empirical study, especially among Chinese nurses. Furthermore, whether the organizational commitment can mediate the effect of workplace bullying on turnover intention remains unclear.

### Theoretical framework and research hypotheses

Conservation of resources (COR) theory is one of the most classic theories in the organizational behaviour literature [30]. COR theory holds the tenet that individuals have a tendency to endeavour to acquire, maintain, cultivate and protect the resources they value, it comprehends and predicts work-related stress. These valuable resources include social bonds, energy and personal power [31]. COR suggests that workplace bullying is a stressful stressor and may put nurses in a state of loss or depletion of resources, but the effects of this may be moderated by individual difference variables. Nurses’ accumulation of resources can protect or renew additional resources, which reduces the level of turnover intention [32], and if resources are difficult to restore in a timely manner, this may trigger negative work attitudes and behaviours in nurses [33]. According to the Social Exchange Theory, based on the principle of reciprocity, the organization provides working conditions and remuneration, and employees develop a sense of obligation to give o the organization in order to maintain the stability of the exchange relationship with the organization. Organizational commitment is a social exchange relationship between an individual and the organization [34]. Organizational commitment is a key resource and previous studies have highlighted and demonstrated the moderating role of organizational commitment in influencing employees’ behaviours, such as turnover behavior [35–37].

**Fig. 1:**
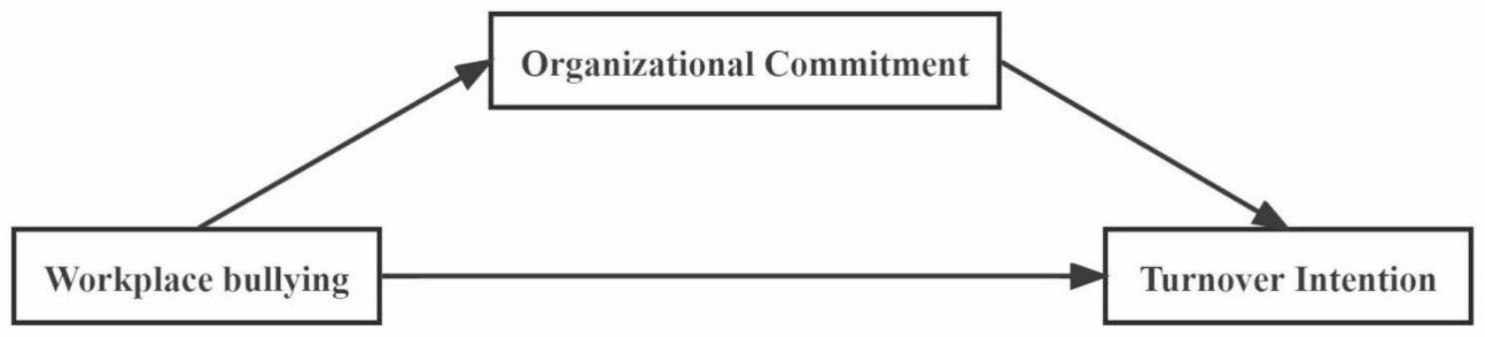
Hypothetical relationship diagram

By integrating the COR Theory and the Social Exchange Theory, it appears plausible to assume self-efficacy would positively moderate the relationship between workplace bullying and turnover intention. The setting of this mediating relationship is crucial in revealing the mechanisms by which workplace bullying affects an individual’s turnover intention. Using organizational commitment as an intermediary may deepen our understanding of how workplace bullying affects nurses’ turnover intention. Therefore, this study aims to determine the mediating role of organizational commitment in the relationship between workplace bullying and turnover intention of clinical nurses in China. As shown in Fig. 1, we established a theoretical hypothesis model and proposed the following three hypotheses: (1)workplace bullying is negatively related to organizational commitment and positively related to turnover intention; (2) organizational commitment is negatively related to turnover intention; and (3)organizational commitment mediates the relationship between workplace bullying and turnover intention.

## Materials and methods

### Study design

This study examined the relationship between workplace bullying, organizational commitment and turnover intention among clinical nurses using a descriptive cross-sectional design.

### Participant enrollment

A convenient sampling method was used in this cross-sectional study. Clinical nurses were recruited from hospitals in 26 provincial administrative regions in China, from December 2, 2021 to February 25, 2023.

The inclusion criteria of the study were as follows: (1) nurses have been registered, (2) nurses are currently engaged in clinical practice and more than six months of clinical work experience, and (3) nurses are volunteered for the study. Nurses who are interns, or who have participated in other relevant studies were excluded from the study.

Kline recommends that the sample size for structural equation modelling (SEM) should exceed 200 [38]. A widely used priori sample size calculator, which is openly designed for calculating SEM sample sizes was applied (https://www.danielsoper.com/statcalc/calculator.aspx?id=89). Moderate effect = 0.3, power value = 0.95, α = 0.01 and including 3 latent and 10 observed variables, the minimum sample size for this study is 237. A total of 750 nurses participated in the survey, but 155 unqualified questionnaires were deleted due to answering time of less than 180 s or highly repetitive answers. Consequently, 585 valid questionnaires were included in the analyses (valid response rate = 78.0%), which met the aforementioned sample size requirements.

### Data collection

We have set up an electronic questionnaire a Chinese free online platform named Wenjuanxing (https://www.wjx.cn/), and sent it as a link or Quick Response code to nurses who met the inclusion criteria. Wenjuanxing is a relatively safe platform, without any risk of data being damaged or leaked by third parties. Since the participants are Chinese clinical nurses, the language of the questionnaire is set to Chinese. In order to improve the response rate of nurses, the research team designed a beautiful electronic questionnaire poster. As members of the Chinese Nursing Association, the researchers sent a recruitment notice in the WeChat group of the Chinese Nursing Association members. The first page of the e-questionnaire contained the informed consent form and states that the e-questionnaire is anonymous and does not contain any identifying information, and only participants who signed the informed consent form were allowed to proceed to the next page to complete the questionnaire. The questionnaire could only be submitted after it had been completed and each IP address could only answer once to avoid repeated answers. Researchers directly exported the data from Wenjuanxing to avoid possible errors in manual data entry. After confirming that the data is undoubtedly correct, the data administrator locked the database to prevent data leakage.

### Measurement

***The Socio-demographic Questionnaire*** was self-administered and was designed to collect the demographic characteristics of participants such as age, gender, educational level, years of nursing experience, working department and professional title.

***Negative Acts Questionnaire (Chinese version; NAQ)*** was used to assess workplace bullying. The original version was developed by Einarsen and Raknes [39] in 1997 and translated into Chinese and revised by Jiang [40]. The Chinese version of NAQ has been widely used in research on workplace bullying. The NAQ consists of 20 items: 11 on personal bullying, 9 on work-related bullying. Each item is evaluated on a four-point Likert scale (1 = “never” to 4 = “very often”) and a higher score indicates a more severe level of workplace bullying. A total score lower than 33 points indicates no bullying, scores between 23 and 41 indicate frequent bullying, and more than 45 points indicates serious bullying. The Cronbach’s alpha for the scale was 0.91 and the retest reliability was 0.73 [40].

***The Chinese Workers’ Organizational Commitment Scale (QCQ)*** was developed by Ling [41]. It is the most widely used questionnaire to measure the organizational commitment of participants. The scale consists of five dimensions with 25 items: emotional commitment, normative commitment, aspirational commitment, economic commitment and opportunity commitment. All items are measured on a Likert five-point scale from 1 (strongly disagree) to 5 (strongly agree). The score for each dimension is the sum of the items and ranged from 1 to 25, and the total scale score was the sum of the dimensions and ranged from 25 to 125, with higher scores representing higher levels of organizational commitment. The Cronbach’s alpha for the scale and each dimension were between 0.67 and 0.85, and the retest reliability were between 0.72 and 0.89 [41].

***Turnover Intention Questionnaire (Chinese version; TIQ)*** aimed to measure participants’ turnover intention. Michael and Spectpr [42] developed the original version and Lee translated it into Chinese [43]. The six-item scale is divided into three dimensions: the possibility of employees quitting their present job, the motivation for employees to seek other jobs and the possibility of employees having access to external job. Participants respond on a four-point Likert scale (1 = “never” to 4 = “often”) and a higher average score indicating a stronger intention to leave. A total average score lower than 1 point indicates turnover intention is very low, scores between 1 and 2 indicate low, high when it is between 2 and 3, and very high when it is greater than 3. The Cronbach’s alpha for the scale in this study was 0.77 and the content validity index is 0.68 [43]. This scale has demonstrated good reliability and validity and has been widely used in nurse.

### Ethical considerations

The ethics approval was obtained from the ethics committee of Shenzhen Hospital of Southern Medical University and Xiangya School of Stomatology, Central South University [No: 20,220,109]. Prior to filling out questionnaires, all eligible participants were informed of the purpose of the study and signed an informed consent form, which informed that the entire study process was conducted completely voluntarily, anonymously and confidentially, and they had the right to decline or withdraw from the study at any time.

### Data analysis

First, descriptive data, including means and standard deviations, frequency, and percentage, were used to describe the demographic information, workplace bullying, organizational commitment and turnover intention. Pearson’s correlation was used to examine the correlations between variables. Descriptive statistics and Pearson’s correlation were conducted using IBM SPSS 23.0. P-values < 0.05 were considered statistically significant (2-sided tests).

Second, SEM was computed with AMOS 23.0 to test the hypothesized model with the maximum likelihood estimation methods. The input for each analysis was the covariance matrix of the items. The goodness-of-fit of the model was evaluated using using absolute and relative indices. The absolute indices including χ2/df and Root Mean Square Error of Approximation (RMSEA). If the χ2/df was < 3, the model would be regarded as a good fit; if it was between 3 and 5, the model would be considered an acceptable fit. Furthermore, if the RMSEA value was < 0.05, the model would be regarded as a good fit, and if the value was < 0.08, the model would be counted as a reasonable fit. The relative indices including Comparative Fit Index (CFI), Goodness-of-Fit Index (GFI), Normed Fit Index (NFI), Relative Fit Index (RFI), Tacker-Lewis Index (TLI). If the CFI, GFI, NFI, RFI and TLI values were > 0.90, it would indicate that the model achieved a good fit [44].

## Results

### Sample characteristics

A total of 750 nurses participated in the survey, 155 nurses were excluded due to answering time of less than 180 s or highly repetitive answers. Consequently, only 585 nurses were included in the analyses (valid response rate = 78.0%). Most nurses were female (86.8%), age was 29.63 ± 5.68 (years), and participants represented many specialty departments. The other socio-demographic characteristics of the nurses are shown in Table 1.

**Table 1:** Socio-demographic characteristics of participants (N = 585)

Variables	Category	n	%
**Gender**	Male	77	13.2
	Female	508	86.8
**Age (years)**	≤ 25	141	24.1
	26–35	365	62.4
	≥ 36	79	13.5
**Education level**	Associate degree	115	19.7
	Bachelor degree	441	75.3
	Master degree or above	29	5.0
**Department**	Medical	191	32.6
	Surgical	207	35.4
	Emergency or ICU	88	15.1
	Others	99	16.9
**Years of nursing experience**	≤ 1 years	33	5.6
	2–5 years	243	41.6
	6–10 years	148	25.3
	11–20 years	89	15.2
	> 30 years	72	12.3
**Professional title**	Junior RN	399	68.2
	Supervisor nurse	165	28.2
	Associate professor or professor nurses	21	3.6
**Employment type**	Formal employed nurse	492	84.1
	Personal agent nurse	93	15.9

### Descriptive analysis of workplace bullying, organizational commitment and turnover intention

As shown in Table 2, the average total scores of workplace bullying, organizational commitment and turnover intention were 38.55 (SD 15.69), 71.69 (SD 17.55), and 16.94 (SD 3.70), respectively. 17.3% of nurses reported they have experienced bullying and 37.1% reported severe bullying, 79.5% reported average or high levels of organizational commitment, and 86.4% reported high or very high levels of turnover intention. The detailed descriptive results are shown in Table 3.

**Table 2:** Descriptive Analysis of Workplace Bullying, Organizational Commitment and Turnover Intention(i) (N = 585)

Variables		Mean ± SD	Total scores	Average score of items	
**Workplace Bullying**	personal bullying	20.90 ± 8.52	38.55 ± 15.69	1.93 ± 0.78	
	work-related bullying	17.65 ± 7.58			
**Organizational Commitment**	emotional commitment	14.82 ± 4.54	71.69 ± 17.55	2.86 ± 0.70	
	normative commitment	15.25 ± 4.43			
	aspirational commitment	15.22 ± 4.36			
	economic commitment	13.54 ± 4.16			
	opportunity commitment	12.87 ± 3.79			
**Turnover Intention**	the possibility of employees quitting their present job	5.40 ± 1.64	16.94 ± 3.70	2.82 ± 0.62	
	the motivation for employees to seek other jobs	5.31 ± 1.69			
	the possibility of employees having access to external job	6.22 ± 1.19			

**Table 3:** Descriptive Analysis of Workplace Bullying, Organizational Commitment and Turnover Intention(ii) (N = 585)

Variables	level	n	%
**Workplace Bullying**	Not bullied	267	45.6
	Experienced bullying	101	17.3
	Experienced severe bullying	217	37.1
**Organizational Commitment**	Very low	1	0.2
	Low	92	15.7
	Medium	225	38.5
	High	240	41.0
	Very high	27	4.6
**Turnover Intention**	Very low	2	0.3
	Low	78	13.3
	High	280	47.9
	Very high	225	38.5

### Correlation between workplace bullying, organizational commitment and turnover intention

Table 4 shows that each factor of workplace bullying was significantly negatively correlated with the total score of organizational commitment and each dimension, significantly positively correlated with the total score of turnover intention and each dimension. Additionally, that each factor of organizational commitment had significant positive correlation with the total score of turnover intention and each dimension.

### Mediating role of Organizational Commitment on Workplace bullying and turnover intention

The initial hypothetical model (Fig. 1) showed unsatisfactory fit. Then, based on the modification indices, the correlations between some residuals of the observed variables should be added, such as e1 ↔ e3, e4 ↔ e8, and so on. As shown in Table 5, the final model indicated a good fitting effect. Table 6 displays the maximum likelihood estimate of the modified model. The χ^2^/df ratio was 2.792 (χ^2^ = 69.801, df = 25), and the RMSEA was 0.055. Furthermore, the CFI, GFI, NFI, RFI, and TLI values were higher than 0.900 (CFI = 0.989, GFI = 0.978, NFI = 0.984,RFI = 0.971, and TLI = 0.981).

**Fig. 2:**
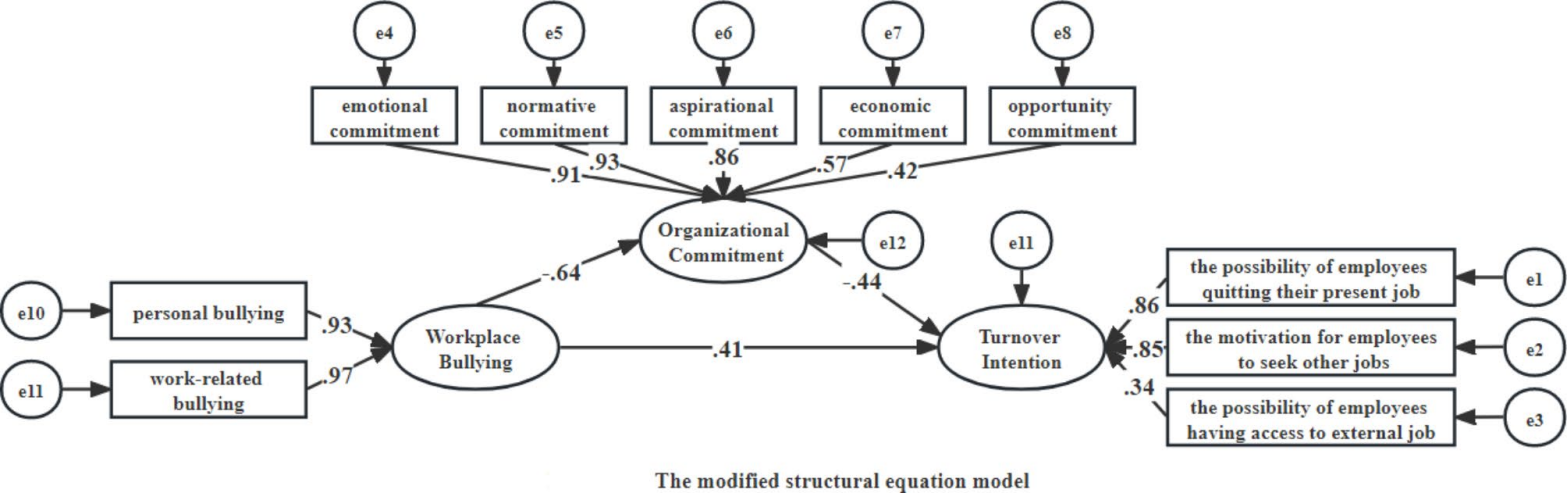
The modified structural equation model

As shown in Fig. 2, workplace bullying could negatively predict organizational commitment (β = −0.64, P < 0.001) and positively predict turnover intention (β = 0.41, P < 0.001), organizational commitment could negatively predict turnover intention(β = −0.44, P < 0.001). Further, organizational commitment partially mediated the relationship between workplace bullying and turnover intention, and the total mediating effect was (− 0.641) × (− 0.437) = 0.280; the total effect was 0.41 + 0.28 = 0.69. The mediation effect amount was 40.58%.

**Table 4:** Correlation matrix for workplace bullying, organizational commitment and turnover intention

	*Variables*	1	2	3	4	5	6	7	8	9	10	11	12	13
1	Personal bullying	1												
2	work-related bullying	0.897*	1											
3	emotional commitment	-0.445*	-0.487*	1										
4	normative commitment	-0.549*	-0.564*	0.850*	1									
5	aspirational commitment	-0.550*	-0.576*	0.761*	0.797*	1								
6	economic commitment	-0.266*	-0.300*	0.509*	0.529*	0.539*	1							
7	opportunity commitment	-0.129*	-0.163*	0.430*	0.392*	0.395*	0.704*	1						
8	the possibility of employees quitting their present job	0.516*	0.553*	-0.578*	-0.563*	-0.541*	-0.302*	-0.154*	1					
9	the motivation for employees to seek other jobs	0.552*	0.580*	-0.534*	-0.529*	-0.493*	-0.274*	-0.148*	0.735*	1				
10	the possibility of employees having access to external job	0.131*	0.167*	-0.224*	-0.170*	-0.169*	-0.166*	-0.274*	0.365*	0.290*	1			
11	TWB	0.977*	0.971*	-0.477*	-0.571*	-0.577*	-0.290*	-0.149*	0.548*	0.580*	-0.152*	1		
12	TOC	-0.482*	-0.519*	0.877*	0.881*	0.860*	0.788*	0.692*	-0.531*	-0.491*	0.242*	-0.512*	1	
13	TTI	0.525*	0.565*	-0.573*	-0.547*	-0.520*	-0.313*	-0.224*	0.899*	0.878*	0.617*	0.558*	-0.539*	1

TWB, Total workplace bullying; TOC, total organizational commitment; TTI, total turnover intention

*P < 0.01

**Table 5:** Correlation matrix for workplace bullying, organizational commitment and turnover intention

Model	χ^2^/df	P	CFI	GFI	NFI	RFI	TLI	RMSEA
Initial model	14.207	<0.001	0.900	0.878	0.894	0.851	0.860	0.150
Revised Model	2.792	<0.001	0.989	0.978	0.984	0.971	0.981	0.055

**Table 6:** Maximum likelihood estimates of the modified model

Pathway	Standardized coefficients	Standard errors	Critical ratio	*P*
Workplace Bullying→Organizational Commitment	−0.641	0.021	-17.000	< 0.001
Organizational Commitment→Turnover Intention	−0.437	0.016	-9.260	< 0.001
Workplace Bullying→Turnover Intention	0.407	0.009	8.561	< 0.001

## Discussion

This study aimed to explore the role of organizational commitment between workplace bullying and turnover intention among clinical nurses in China. The findings of this study provide valuable insights into reducing nurses’ turnover intention. We identified that workplace bullying was negatively correlated with the organizational commitment and positively correlated with turnover intention, and there is a negatively correlation between organizational commitment and turnover intention. More importantly, organizational commitment played a partial mediating role in the relationship between workplace bullying and turnover intention, which elucidated the underlying psychological mechanisms and facilitated a scientific foundation for developing targeted interventions.

### Prevalence of workplace bullying among chinese clinical nurse

As a source of work–family conflict, workplace bullying is common among nurses and has a non-negligible impact on nurses’ work and life [45–47]. Our research showed that the prevalence of workplace violence among nurses (54.6%) is relatively lower than the findings of Zhang (68.31%) [6] ,and higher than Chen’s results (46.2%) [48] and Fei’s results (41.99%) [49]. This difference may be related to differences instruments and the different percentages of nurses with different work experience. Previous study have proposed that experienced nurses were better able to deal with clinical issues and interpersonal relationships [50], whereas only 27.5% of the nurses had more than 10 years of experience and a higher percentage of nurses who had less than 5 years of experience among the participants in the present study (47.2%). The relatively low position of young nurses and their job performance reflecting a need for further skill may lead to more frequent workplace bullying [1].

### Workplace bullying is negatively correlated with organizational commitment and positively correlated with turnover intention

Our research showed workplace bullying was negatively related to organizational commitment and positively related to turnover intention, which supported hypothesis 1 and was consistent with the findings of Kang [51], Qi [52] and Ren [53]. Workplace bullying leads to a range of negative effects, both on the individual (physical and mental health, post-traumatic stress disorder, etc.) and on the organization (interpersonal relationships, organizational performance, etc.). Strasser and Bateman [54] point out that organizational commitment is a multi-faceted phenomenon mainly related to employees’ feelings towards the organization. Our study showed that employees who perceived themselves to be bullied had significantly lower levels of organizational commitment, which is similar to the findings of Rodwell J et al. [55]. In other words, when nurses experience workplace bullying, their desire for additional organizational work, the degree of organizational goals, value alignment, and organization-related aspirations are greatly reduced, i.e. organizational commitment is reduced. Besides, Al Muharraq et al. [56] found that workplace bullying is one of the main reasons for high attrition and low retention rates in hospitals, and its frequency is one of the best predictors of turnover intention. A qualitative systematic review pointed that nurses resigned to “protect their sanity” and believed that turnover was the only solution to bullying [57], and Wolf ‘s study also showed that nurses would choose to leave in order to escape bullying [58]. There is no doubt that the departure of nurses as a result of workplace bullying is a huge loss to healthcare organizations. Therefore, it is essential to tackle the turnover intention resulting from bullying experience.

### Organizational commitment is negatively correlated with turnover intention

Besides, we found organizational commitment is negatively related to turnover intention, which supported hypothesis 2 and was consistent with Neves T [59] and Callado A’s study of Portuguese nurses [60] and Labrague’s study of Filipino nurses [61]. The five dimensions of organizational commitment are negatively correlated with turnover intention. Specifically, nurses with high emotional commitment tend to be more loyal to the hospital and invest more to make themselves and the hospital become a community. Nurses with high normative commitment can abide by professional norms, implement industry standards and be loyal to the nursing career. The high aspirational commitment of nurses means that they have a large space for career development and the high economic commitment means that nurses have good salary and welfare treatment. Nurses with a high opportunity commitment will consider the financial consequences of quitting their current job and whether they can find a more suitable job. Therefore, nurses with high organizational commitment tend to have low turnover intention.

### Mediating role of Organizational Commitment on Workplace bullying and turnover intention

Our study confirmed that organizational commitment can partially mediate the effect of workplace bullying on turnover intention, which supported hypothesis 3. Some researchers have highlighted the need for empirical assessments and theoretical explanations of mediating and moderating variables that can explain the effects of bullying [10, 25], the effects of self-efficacy and work stress have been confirmed by Hsieh [62] and Malik [63], obviously, most of the proven factors that can mediate workplace bullying and turnover intention were psychological factors of nurses. Our findings complement this research topic and showed that nurses who experience workplace bullying may further reduce organizational commitment, which increases turnover intention. Studies by Yang [64] and Lee [65] also noted the importance of increased organizational commitment in reducing nurses’ turnover intention.

The COR theory pointed that individuals have limited resources within a specific period, and when resources are lost, they need to acquire resources from the external environment. Nurses invest a significant amount of resources, such as time, knowledge, empathy, and technical skills, into their work environment. Workplace bullying can lead to a higher depletion of their psychological resources, accelerating resource loss. Nurses who were bullied in the workplace environment have a lower sense of belonging to the organization, and may have a desire to leave and negative behavior at work. According to the Social Exchange Theory, when nurses are relatively satisfied with the resources provided by the organization, they reciprocate by demonstrating organizational commitment and working with a more positive attitude, which can to some extent reduce nurses’ turnover intention.Conversely, workplace bullying has a negative effect on organizational commitment, and the more workplace bullying nurses experience, the lower organizational commitment. When the nurses feel that the feedback from the organization is reduced, they will gradually entrust the need for interpersonal relationship and belonging to the next organization, and thus have a stronger idea of changing jobs in their hearts. Based on our research findings, in order to mitigate the negative effects of workplace bullying, reduce nurses’ turnover intention, and stabilize the nursing workforce, we can focus on We can focus on reducing the incidence of workplace bullying and improving the level of nurses’ organizational commitment.

Firstly, there is an urgent need for measures to reduce nurses’ turnover intention by directly reducing the incidence of workplace bullying. Filipova A. A’s findings suggest that healthcare facilities that focus on addressing bullying issues, may be good for improving nurse commitment and retention [29]. Park’ [4] research demonstrated that the simulation-based learning can improve awareness of workplace bullying and develop coping strategies for the same. Griffin’s cognitive rehearsal program had a positive effect on workplace bullying prevention programs [66]. This suggests that managers should step up efforts to publicize the harm of workplace bullying, strengthen nurses’ awareness of workplace bullying, and set a model of appropriate behavior for employees after being properly trained, such as violence prevention skills training, so that nurses can recognize and resist workplace bullying in time [67]. Besides, in order to reduce workplace bullying among nurses, hospital managers should improve the relationship-oriented organizational culture and mitigate the hierarchy-oriented culture [68], and enacting relevant institutional regulations, such as emphasizing in the “Code of Ethics for Nurses” that nurses should maintain a collaborative and respectful relationship with colleagues, and oppose lateral violence in the workplace. It was found that having a system for reporting all bullying incidents leads to significantly lower levels of bullying [69], so leadership can require specific anti-bullying language in organizational policies and establish a credible set of policies and procedures and multiple channels to support those accused of bullying (e.g., confidential multi-assessment 360° assessments) [70].

Secondly, it is necessary to improve the level of nurses’ organizational commitment to reduce the impact of work bullying on turnover intention. The pay-return imbalance model discusses the mechanism of work stress from the perspective of social exchange theory. The theory is that the time and energy people put into their jobs needs to be compensated by money, respect, and opportunities for professional development. Once the organization fails to give corresponding rewards to the employees, the employees will change their original work status, such as late arrival and early departure, absenteeism, decreased satisfaction, job burnout, and reduced organizational commitment [71]. The results of this study indicate that nurses exhibited relatively lower levels of commitment in the dimensions of economic and opportunity commitment among the five dimensions of organizational commitment. Therefore, we recommend that nursing managers prioritize these two dimensions and take targeted measures to improve their levels of organizational commitment. On the one hand, it is necessary to improve the organizational culture and environment of the system to solve the root problem of workplace bullying, managers should establish a fair performance management system and a reasonable incentive system [72, 73], practical strategies could include offering competitive employee benefits, establishing employee-employer relationships, involving them in their own performance appraisal process, etc. [74]. These measures can help nurses recognize that their efforts can be rewarded accordingly, thereby contributing to an improvement in their levels of economic commitment. On the other hand, it is important to establish a fair and well-defined promotion mechanism that offers nurses a transparent and viable career development path with ample opportunities for growth. Besides, offering additional learning opportunities can contribute to enhancing nurses’ levels of opportunity commitment. Albooghobeish’s research suggests that professional ethics education based on a multi-method approach can increase nurses’ organizational commitment [75]. By creating an environment that promotes continuous learning and development, nurses are more likely to perceive a promising career trajectory and remain dedicated to the organization. Furthermore, hospital administrators can enhance nurses’ overall organizational commitment by employing long-term commitment strategies to retain experienced employees, constructing an empowering work environment, implementing flexible work systems, fostering a positive organizational culture, and strengthening the development of a magnetic hospital [76–78]. By implementing these measures, administrators can foster a sense of loyalty and attachment among nurses towards the organization, consequently enhancing their organizational commitment.

#### Limitations

This study has several limitations. First of all, convenience sampling was adopted in this study due to practical reasons such as resource constraints, convenience sampling is a non-probability sampling method that relies on individuals who are easily accessible or readily available to participate in the study, which may lead to selection bias. Future studies should consider using more representative sampling methods to increase the generality of the findings. Second, the participants in this study were mainly recruited from hospitals in central and southern China through convenience sampling, and the proportion of nurses with less than 5 years of experience was close to average, which may generate selection bias and limit the generality of the findings. Future studies should consider expanding the geographical range of the sample and balancing the selection of participants from different regions and different working years. Third, despite the researchers’ diligent efforts to interpret the questionnaire prior to collection, our data relied solely on self-reported responses and may lead to information bias. Therefore, data from care administrators, physicians, and patients can be collected in the future to further complement and enhance our research. Fourth, although SEM is generally referred to demonstrate the direct and indirect associations of variables, the lack of use of longitudinal data prevents the interpretation form reflecting true causality. Therefore, further longitudinal studies should be conducted to better explore the long-term and dynamic effects of workplace bullying on turnover intention of nurses.

### Practical implications of the study

Workplace bullying should be addressed at both organizational and unit levels. By recognizing workplace bullying is a key factor influencing nurses’ intention to leave, healthcare organizations can be motivated to develop and implement comprehensive anti-bullying policies and procedures. In addition, healthcare organizations should invest in educational programs and training initiatives to raise awareness among healthcare workers about the detrimental effects of workplace bullying and promote a culture of respect and collegiality in order to create a supportive and respectful work environment.

Our findings also highlight the importance of organizational commitment, which suggests that nursing managers should pay attention to enhancing the nurses’ organizational commitment, encouraging and supporting nurses to work adequately, and improving their sense of identification with the organization in order to achieve stability in the nursing workforce.

## Conclusion

Our findings demonstrated that organizational commitment served as a mediator between workplace bullying and turnover intention. Healthcare organizations and nursing managers should develop appropriate strategies and actively carry out relevant continuous education and training to enhance nurses’ organizational commitment in order to reduce their turnover intention and stabilize the nursing workforce.

## Data Availability

All methods were carried out in accordance with academic standard and declaration of Helsinki. All relevant data are within the manuscript and its Additional files.
