# Correction to: The mediating role of organizational commitment between workplace bullying and turnover intention among clinical nurses in China: a cross-sectional study

**DOI:** 10.1186/s12912-023-01591-4

**Published:** 2023-11-20

**Authors:** Guili Xia, Yi Zhang, Ling Dong, Fengtao Huang, Yao Pu, Jiang Luo, Yi-ping Chen, Zhengxia Lei

**Affiliations:** 1 Department of Gastroenterology, Shenzhen Hospital, Southern Medical University, Shenzhen, Guangdong China; 2 Hunan Key Laboratory of Oral Health Research & Xiangya Stomatological Hospital & Xiangya School of Stomatology, Central South University, Changsha, Hunan 410008 China

**Correction to: BMC nursing (2023) 22:360**

10.1186/s12912-023-01547-8

Following publication of the original article [1], the authors reported errors in references 22, 31 and 57. They need to be updated.

The original version was:

22. Sir de Sul LP. Translation and validation of the anticipated turnover scale for the Portuguese Cultural Context. Nurs Open. 2020;7(5):1475–81. 10.1002/nop2.521.

31. Usman M, Ali M, Anwar F, Khan MAS. The relationship between laissez-faire leadership and burnout: mediation through work alienation and the moderating role of political skill. Can J Adm Sci / Revue Canadienne des Sciences de l’Administration. 2020;37(4):423–34. 10.1002/cjas.1568.

57. Shorey S, Wong PA, Qualitative Systematic. Review on Nurses’ Experiences of Workplace bullying and implications for nursing practice. J Adv Nurs. 2021;77(11):4306–20. 10.1111/jan.14912.

The updated version is:

22. De Sul SIR, Lucas PRMB. Translation and validation of the anticipated turnover scale for the Portuguese Cultural Context. Nurs Open. 2020;7(5):1475–81. 10.1002/nop2.521.

31. Usman M, Ali M, Yousaf Z, Anwar F, Waqas M, Khan MAS. The relationship between laissez-faire leadership and burnout: mediation through work alienation and the moderating role of political skill. Can J Adm Sci / Revue Canadienne des Sciences de L’ Administration. 2020;37(4):423 − 34. 10.1002/cjas.1568.

57. Shorey S, Wong PZE. A qualitative systematic review on nurses’ experiences of workplace bullying and implications for nursing practice. J Adv Nurs. 2021;77(11):4306-20. 10.1111/jan.14912.

The original article [1] has been corrected.
